# Comparative Transcriptomics Reveals Gene Families Associated with Predatory Behavior in *Photuris femme fatale* Fireflies

**DOI:** 10.3390/genes11060627

**Published:** 2020-06-07

**Authors:** Cheyenne N. McKinley, Sarah E. Lower

**Affiliations:** Department of Biology, Bucknell University, Lewisburg, PA 17837, USA; cnm006@bucknell.edu

**Keywords:** molecular evolution, dN/dS, *Photinus*, FUSTr

## Abstract

Identifying the basis of phenotypic variation is a key objective of genetics. This work has been mostly limited to model systems with a plethora of genetic manipulation and functional characterization tools. With the development of high-throughput sequencing and new computational tools, it is possible to identify candidate genes related to phenotypic variation in non-model organisms. Fireflies are excellent for studying phenotypic variation because of their diverse and well-characterized behaviors. Most adult fireflies emit a single mating flash pattern and do not eat. In contrast, adult females of many species in the genus *Photuris* employ multiple flash patterns and prey upon mate-seeking males of other firefly species. To investigate the genetic basis for this variation, we used comparative transcriptomics to identify positively selected genes between a predatory firefly, *Photuris* sp., and a non-predatory relative, *Photuris frontalis*, controlling for genes generally under selection in fireflies by comparing to a *Photinus* firefly. Nine gene families were identified under positive selection in the predatory versus non-predatory *Photuris* comparison, including genes involved in digestion, detoxification, vision, reproduction, and neural processes. These results generate intriguing hypotheses about the genetic basis for insect behavior and highlight the utility of comparative transcriptomic tools to investigate complex behaviors in non-model systems.

## 1. Introduction

The extent to which phenotypes are a result of genetic versus environmental factors is a longstanding question in genetics. Behavior is a particularly intriguing phenotype due to its variability, complexity, and essential functions in organism fitness. What are the identities of genes contributing to this variation? Classical forward and reverse genetics approaches have identified pathways underlying key behaviors such as aggression [1], circadian rhythm [2], and courtship [3]. Other approaches have utilized genetic crosses to map quantitative traits to genomic regions, or leverage natural variation and genome sequence data to investigate the genetic basis of quantitative traits (Quantitative Trait Locus mapping and Genome Wide Association studies, reviewed in [4]). While these techniques are powerful, they may require a combination of genomic data, genetic manipulation tools, large sample sizes, and extensive crosses, thus limiting them to model systems.

To determine how general these findings from model systems are across organismal diversity, new tools have been developed to study the specific genes involved in behavior in non-model systems. One such strategy to identify candidate genes is to use a comparative genomics approach, examining the evolution of gene sequences between close relatives with phenotypic divergence in the trait of interest. Genes with molecular signatures of divergent selection, as compared to the rest of the genome, are candidates for involvement with the phenotype [5,6,7]. One signature is increased rates of nucleotide substitutions that are predicted to cause functional changes in the resulting protein, indicated by elevated rates of nonsynonymous substitutions as compared to rates of synonymous substitutions (dN/dS) [8]. Because this approach is based on sequence data, it can be used in non-model systems where lab culture, genetic manipulation tools, and large sample sizes are difficult to impossible to achieve. Importantly, reference genomes are not a prerequisite for this approach—examining coding sequences genome wide via an assembled transcriptome is sufficient to identify genes with elevated dN/dS [9,10,11]. For example, a comparative transcriptomic approach identified candidate genes related to songbird behavior, including *DPM1*, a gene involved in axonogenesis and eating behavior [9]. Because behaviors are often complex and extremely variable within and across taxa, identifying candidate genes is not a trivial task. One strategy to mitigate this is to study a system with a well-defined behavior.

Fireflies (Coleoptera: Lampyridae) fit this criterion, with well-characterized stereotyped behaviors and variation in these behaviors across species. Furthermore, growing genomic resources make fireflies an excellent non-model system in which to study the genetic basis of behavior [12,13,14,15,16]. In North American species, nocturnal males flash species-specific bioluminescent signal patterns to females in the vegetation. The female then replies with a species-specific flash response, and the pair continue to communicate until the male locates the female and they mate [17]. In the genus *Photinus*, each species has a single species-specific mating flash pattern in its repertoire and differences in these patterns are hypothesized as the primary mechanism for prezygotic reproductive isolation between species [18]. Because flash patterns are generally diagnostic to species, they are used as the primary identification of an individual to species in the field.

In contrast to other North American fireflies, many species in the genus *Photuris* have multiple flash patterns in their repertoire, making them difficult to identify with flash pattern data. Notably, while the adults of most firefly species do not eat [19], *Photuris* are known for their predatory nature. Termed *femme fatales*, *Photuris* females prey upon other fireflies to gain nutrients as well as to sequester prey-produced cardiotoxins (lucibufagins) into their eggs as a chemical defense [20]. Male *Photuris* will eat other fireflies in the lab [21], though there is no direct observational support for male predation in the field. Female *Photuris* predation follows three main strategies—stealing trapped fireflies from spiderwebs (kleptoparasitism) [22], capturing individuals mid-flight (aerial hawking) [23], or luring males of other species by mimicking their species-specific female response flashes (aggressive mimicry) [24]. In lab studies using reared and wild-caught individuals, virgin female *Photuris* respond to their species-specific mating signal, then switch to primarily responding to prey species flash patterns 24 to 72 hours after successful mating [25]. Thus, female *Photuris* not only recognize and produce their own species-specific mating flash pattern, but also mimic prey-species flash patterns, depending on their *femme fatale* status. This behavior likely has a genetic basis as there is no parental care in fireflies, and lab-reared *Photuris* offspring exhibit the behavior [25]. Because these predatory behaviors are highly dependent on vision, recognition and production of prey species flash patterns, and the ability to digest and process toxins, genes involved in these processes may contribute to the complex *femme fatale* phenotype and are predicted to be under positive selection between predatory and non-predatory species. 

Not all *Photuris* species exhibit predatory behavior to the same degree. The genus is divided into two major groups—the *versicolor* group (17 species) and the *frontalis* group (four species), based on morphology and predatory status [19,23,26,27]. Species in the *versicolor* group are voracious predators with complex flash repertoires and virtually identical morphology, making them difficult to identify to species, while those in the *frontalis* group are not predatory and have distinct flash patterns [19]. For example, male *Photuris frontalis (frontalis* group) flash once every ~0.5 seconds, often synchronizing their flashes [23]. *Pt. frontalis* females respond to this pattern with their species-specific reply. In contrast, *Photuris versicolor* (*versicolor* group) males flash six times in quick succession, pausing for five seconds before flashing another six times [28] *Pt. versicolor* females may be responsive to this pattern, emitting their species-specific reply, or instead, as *femme fatales*, respond primarily to the male flash pattern of their prey species, emitting the corresponding prey female reply. Importantly, transcriptome data are publicly available for one representative of the *versicolor* group, *Photuris* sp. (unidentified to species due to morphological similarity within the *versicolor* group), and one from the *frontalis* group, *Pt. frontalis* [13].

**Figure 1:**
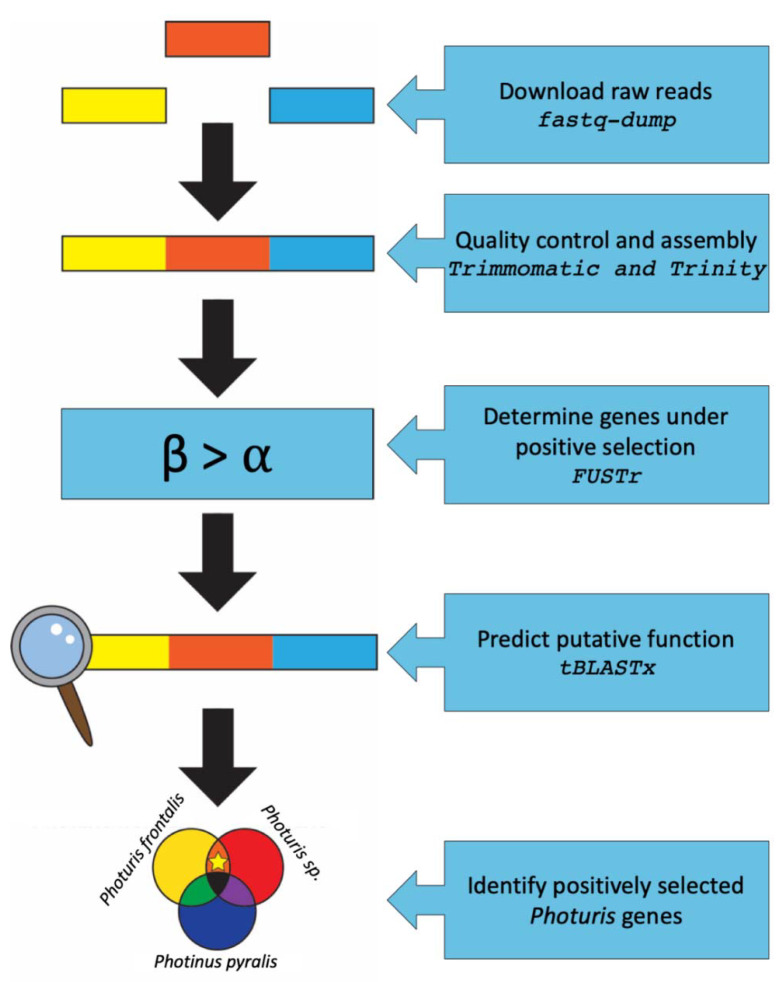
Graphical workflow of computational methods. The specific programs used for each step are shown in italic Courier font. Colored bars represent raw reads that are first downloaded, and then assembled into contigs and quality trimmed. The assemblies are assessed for gene families under positive selection using the posterior probability that β > α. The putative function of the positively selected genes is determined using a homology search. We are interested in the genes involved in predatory behavior, which are in the *Photuris frontalis* and *Photuris* sp. comparison, represented by a star.

In this study, we applied molecular evolutionary analysis on a transcriptome-wide scale to identify candidate genes involved in *Photuris* predatory behavior (Figure 1). To do this, we generated *de novo* transcriptome assemblies from available datasets for three firefly species that differ in their adult feeding behavior (*Photinus pyralis*—non-predatory, *Pt. frontalis*—non-predatory *Photuris*, *Pt.* sp.—predatory *Photuris*). We then used an established computational pipeline to identify gene families under positive selection in pairwise comparisons among the taxa. Finally, we investigated the potential identity and functions of these gene families using sequence homology search strategies. Using this approach, we generated improved assemblies for two species, *Pt. frontalis* and *Pt.* sp., and identified 29 gene families with evidence for positive selection in our pairwise comparisons. Of these gene families, nine were under positive selection only in comparisons between predatory and non-predatory *Photuris* species. Sequence homology searches of these genes revealed their potential involvement in vision, digestion, detoxification, and neural processes.

## 2. Materials and Methods 

### 2.1. Experimental Design

To identify candidate genes involved in predation, we investigated positive selection on coding sequences in pairwise comparisons between three firefly species: *Pt. frontalis*, *Pt.* sp., and *Pn. pyralis*. Genes identified as under positive selection in the within-*Photuris* comparison between *Pt. frontalis* (non-predatory) and *Pt. sp* (predatory) are candidates for involvement in predatory behavior. To rule out genes evolving under positive selection in general in fireflies due to other phenotypic differences, such as a species-specific flash pattern, circadian rhythm, and adaptations to different habitats, we also compared both *Photuris* species with a species outside the genus, *Pn. pyralis* (non-predatory). 

### 2.2. Transcriptome Assembly and Assessment

To ensure that assemblies were compatible with downstream analysis software and to potentially improve published assembly quality, we *de novo* assembled transcriptomes for each species. Briefly, all available paired-end reads for RNA sequencing datasets for each species were downloaded from the NCBI database using fastq-dump from the SRAToolkit v2.9.2 [29]. This included reads derived from a single male’s head tissue for *Pt.* sp., a single male’s head tissue from *Pt. frontalis,* and 30 tissues from multiple males, females, eggs, and larvae for *Pn. pyralis* (Supplementary Table S1) [15,30,31,32]. Reads were then assembled *de novo* using Trinity v2.8.4 [33]. For quality control, the --trimmomatic flag was used to trim the reads, following best practices for *de novo* assembly [34]; otherwise, default parameters were used. 

Because data from only a single tissue from a single sex were available to generate transcriptome assemblies for *Pt.* sp. and *Pt. frontalis*, it is possible that genes involved in predation could be missed if they were not expressed in the sample. To investigate how this limited data might affect our results, we calculated the completeness of our assemblies using Benchmarking Universal Single-Copy Orthologs (BUSCO) v3.3.1 [35], comparing the Trinity assemblies to the endopterygota database of conserved genes. To explore how using different numbers of individuals in the transcriptome assemblies may have affected our results, we also conducted an analysis using only male head data for all three taxa (Supplemental Text 1). Results were qualitatively similar, and thus, we present the results using the more comprehensive dataset with multiple *Pn. pyralis* RNAseq datasets below.

### 2.3. Site-Specific Positive Selection Analysis

To identify genes with evidence for positive selection at amino acid sites, we ran Families Under Selection in Transcriptomes (FUSTr) [36] on each of our three pairwise species comparisons. We chose to use a pipeline that tests amino acid sites rather than averaging over the entire coding sequencing because genes may experience positive selection at specific sites while being conserved overall. In addition, FUSTr has several advantages over existing comparative genomics pipelines: it is fast, handles *de novo* transcriptome assemblies by predicting transcriptomic open reading frames (ORFs), and takes into account isoforms prior to sequence analysis [36]. Briefly, FUSTr uses the engine workflow SnakeMake [37] to automate the following pipeline: it first predicts ORFs using Transdecoder [38], retaining the longest isoform. It then identifies gene families by using the programs BLASTp [39] to detect sequence similarity among ORFs (e-value: 10^−5^), and SiLiX [40] to cluster the gene sequences into putative gene families (groups of homologous genes) based on this similarity. Finally, it detects significant amino acid site-specific positive selection in gene families with 15 or more sequences using Fast Unconstrained Bayesian AppRoximation (FUBAR; [8]). FUBAR uses a Bayesian approach to calculate the posterior probability that a site is under positive selection. Gene families must have a minimum of 15 sequences to achieve the statistical power to detect site-specific selection. A positively selected site will have a posterior probability of > 0.9 for β > ⍺, where β = dN and ⍺ = dS [8]. While FUSTr can also estimate positive selection using likelihood-based methods (i.e., codeml; [41]), we chose to use FUBAR because its approach, averaging over a large number of site classes with unconstrained dN/dS values, has increased statistical power [8]. Because it uses a Markov chain Monte Carlo approach, it can also process large sequencing datasets quickly. 

### 2.4. Functional Annotation

To identify positively selected genes and infer their putative functions, we first extracted and concatenated the individual sequences of the genes in each gene family identified as under selection and concatenated into a single FASTA file for each gene family. We then used these files as queries in a tBLASTx (e-value = 1e^−5^) search against the NCBI nucleotide (nt) database [39].

## 3. Results and Discussion

### 3.1. De Novo Assembly Results in Relatively Complete Transcriptomes for All Three Species

At least 2 Gb of sequence data were used to assemble transcriptomes for each species (Supplementary Table S1). The two resulting *Photuris* assemblies have similar GC contents, as expected for closely-related species (Table 1), and higher BUSCO completeness scores than previously published assemblies (~91% vs. ~88%; Table 2 and Supplemental Table S3), demonstrating improvements in Trinity versions since initial assembly publication [13]. The new *Pn. pyralis* assembly is less complete than the recently published reference gene set (93.5% vs. 95.2%; [15]), likely reflecting differences in assembly methods (e.g., including evidence from HMM models), but is compatible with downstream FUSTr analysis. Even with 44 times the input data (102.5 Gb vs. an average of 2.4 Gb), the *Pn. pyralis* assembly with multiple tissues is only 2.6% more complete than the average *Photuris* assembly (93.5% vs. average 91.1%; Table 2 and Supplemental Table S3). These relatively high completeness scores indicate that we captured the majority of conserved genes in our assemblies. 

### 3.2. Species Comparisons Identify Gene Families under Selection 

Across all comparisons, FUSTr found a total of 1116 genes in 29 families with evidence for positive selection (mean = 38.5 genes per family, range: 15–156). Of these genes, 56.45% had a BLAST hit with an e-value less than 1e^−5^, suggesting that almost half of the genes identified as positively selected are either difficult to detect by BLAST due to extensive divergence at individual domains, or are unique to fireflies. These results indicate that future functional annotation of the firefly specific genes is necessary to understand their role in the divergent processes in fireflies. 

#### 3.2.1. Predatory vs. Non-Predatory *Photuris* Comparison

**Figure 2:**
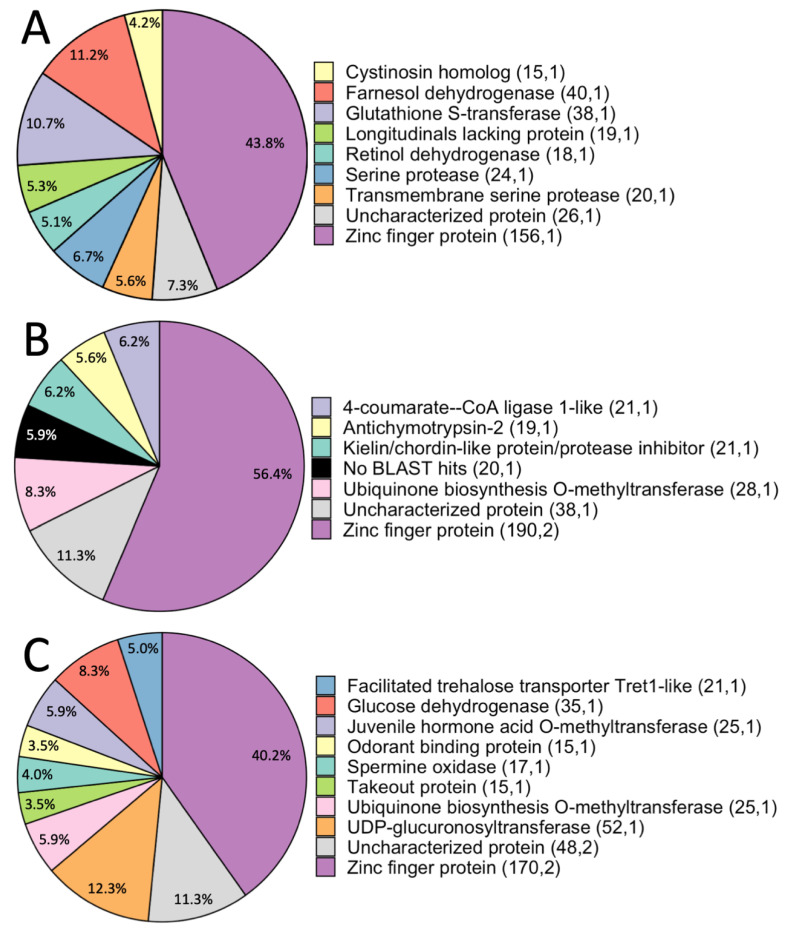
Homologs of genes under positive selection in each comparison identified by BLAST searches. Each color/slice represents the BLAST homolog for each gene family. Percentages represent the number of genes with that functional annotation divided by the total number of genes under positive selection. Parentheticals following each protein name represent (number of genes, number of gene families). (**A**) *Photuris* sp. and *Photuris frontalis* comparison. (**B**) *Photuris frontalis* and *Photinus pyralis* comparison. (**C**) *Photuris* sp. and *Photinus pyralis* comparison. Detailed results, including representation of sequences from each species in the comparison, can be found in supplemental Tables S4–S6.

In the *Pt.* sp. (predatory) versus *Pt. frontalis* (non-predatory) comparison, we identified nine gene families under positive selection (Figure 2). These families represented 20.45% of all gene families analyzed (families with more than 15 sequences) (Table 3). Each gene family was comprised of sequences from both species (Supplementary Table S4). The number of sites with a posterior probability of positive selection > 0.9 ranged from 1–4 sites per family (mean = 2, sd = 1.2 sites per family; Supplemental Table S4). This was greater than the number of sites per family for any other comparisons (predatory *Pt* sp. vs. *Pn. pyralis*: mean = 1.17, sd = 0.4; non-predatory *Pt. frontalis* vs. *Pn. pyralis*: mean = 1.4, sd = 0.52; Supplemental Tables S5 and S6).

##### Genes Involved in Vision, Digestion and Detoxification, and Egg Provisioning Are Candidates for Adaptation to Predation

To successfully prey upon other fireflies, *Pt.* sp. had to evolve to detect, digest, and survive the lucibufagin chemical defenses of their prey. In concordance with our predictions, genes identified as under positive selection in the comparison between predatory and non-predatory *Photuris* are related to these potential adaptations (Figure 2). (i) Vision—retinol dehydrogenases are crucial in creating retinal, a major component of the visual cycle in animals [42]. (ii) Digestion and detoxification—cystinosin homolog and glutathione S-transferases are both involved in digestion [43,44]. In particular glutathione S-transferase is a powerful antioxidant that has been linked to detoxification in other insects, including the model beetle, *Tribolium castaneum*, by increasing the solubility of toxins for easy removal [44].

Female *Photuris* predatory behavior may have evolved not only to gain nutrients, but to acquire prey lucibufagins to provision into their eggs as a chemical defense [45]. Our results suggest a putative mechanism for lucibufagin provisioning. We identified farnesol dehydrogenase, an important component in the production of juvenile hormone (JH) [46] as under positive selection. While JH affects many diverse processes, such as eclosion and metamorphosis, it is a major gonadotropin that regulates mating behavior, oviposition behavior, and vitellogenin synthesis in female insects [47]. Vitellogenin undergoes post-transcriptional processing, which enables it to carry other molecules to the ovaries, where it becomes part of the main nutritional reserves in the egg yolk [47].

##### Juvenile Hormone, Serine Proteases, and *lola* May Be Involved in Neural Processes Underlying Predation

In addition to physiological adaptations to predation, there are also major behavioral components to the *Photuris femme fatale* phenotype—females initially respond to their species-specific mating flash pattern and then switch to aggressive mimicry of prey species’ flash patterns after mating [25]. Because juvenile hormone regulates mating behavior and oviposition behavior, it may also regulate this behavioral switch to aggressive mimicry. Our analysis identified two other gene families with sites under positive selection with potential neural functions, serine proteases, and longitudinals lacking proteins (*lola*). Serine proteases are generally known for their function in digestion, and one midgut-specific serine protease has been identified in the larvae of the firefly *Pyroceoelia rufa*, likely involved in digesting their snail-based larval diet [48]. However, members of this large gene family have been linked to diverse functions including immunity and development, and there is evidence that serine proteases are broadly involved in synaptic function, impacting cognition and behavior, which would be more likely to be found in our head transcriptomes [49,50,51,52,53,54]. Because our data are from head transcriptomes, we do not expect to find midgut-specific proteins, and thus, the serine proteases identified may be involved in neural processes. On the other hand, *lola* is a transcription factor involved in axon guidance and growth [55]. In *Drosophila melanogaster*, *lola* interacts with *fruitless* to affect the number, structure, and function of cells in neural circuits underlying sexually-dimorphic courtship behavior [56]. Future expression and functional studies on *lola* and serine proteases may illuminate their effects on predatory behavior in fireflies.

#### 3.2.2. *Photuris*-*Photinus* Comparisons

We identified 12 gene families with amino acid sites under positive selection in the *Pt.* sp. (predatory) and *Pn. pyralis* (non-predatory) comparison, comprising 24.49% of gene families analyzed (Table 3), a higher percentage than the other comparisons. In contrast, the *Pt. frontalis* (non-predatory) and *Pn. pyralis* (non-predatory) comparison had the lowest number and percentage of gene families under positive selection with eight families, comprising 16.67% of the total families analyzed (Table 3). The higher proportion of gene families under positive selection in the *Pt.* sp.-*Pn. pyralis* comparison could be because these two species are the most phenotypically divergent and are also in different genera. 

##### Diapause, Immunity, and Venom Proteins Are Identified in Non-Predatory Photuris-Photinus Comparisons

In the non-predatory *Pt. frontalis* and *Pn. pyralis* comparison, positively selected genes are involved in other biological processes than predatory behavior (Figure 2). For example, kielin/chordin-like protein has been shown to enhance bone morphogenic protein (BMP) signaling, which has been shown to regulate insect diapause [57,58]. Firefly species emerge at different times over the course of the season, indicating potential differences in diapause regulation. Antichymotrypsin-2 is a protein belonging to the serpin family. Serpins are a superfamily of serine protease inhibitors that are largely involved in innate immunity in insects [59]. 4-coumarate-CoA ligase was also found as under selection, and it has been found to be a venom protein in the parasitoid wasp, *Tetrastichus brontispae.*

##### Candidate Genes Identified in the Predatory *Pt.* sp. and Non-Predatory *Pn. pyralis* Comparison Are Implicated in Both Predation and in Other Phenotypic Variation

Many of the proteins we identified as positively selected in the *Pt.* sp. (predatory) and *Pn. pyralis* (non-predatory) comparison have diverse functions, and may be under positive selection due to other divergent processes than predatory behavior (Figure 2). For example, we found spermine oxidases, part of the polyamine family of proteins, which are involved with cell differentiation, growth, and apoptosis [60,61]. Due to its diversity of function and ubiquity, the identification of spermine oxidases is likely due to general divergent factors. We also identified odorant binding proteins (OBPs) that are crucial in olfaction [62]. However, only one sequence of OBPs was from *Pt.* sp., suggesting that much of the selection detected in this gene family is due to within-*Photinus* sequence evolution. 

However, it is possible that genes involved in predatory behavior may be positively selected, because this is still a predatory versus non-predatory comparison. In one such example, we identified juvenile hormone acid o-methyltransferase (*JHAMT*), which is involved in the late stages of JH synthesis [63]. Again, JH has diverse functions, but is also a major gonadotropin involved in vitellogenin synthesis [47], adding to the support that JH is an important component of predatory behavior in *Pt.* sp.

Also related to reproductive processes, we identified glucose dehydrogenase as positively selected, which catalyzes the reaction from β-D-glucose to δ-gluconolactone [64,65]. This reaction is a necessary step in eclosion in *Drosophila melanogaster* [66]. However, glucose dehydrogenase is also utilized in sperm storage and utilization in the spermatheca of female *Drosophila* [67]. Because aggressive mimicry in *Pt.* sp. only occurs after successful fertilization, sperm storage and utilization may affect this behavior [25].

We also identified Trehalose transporter proteins, which are digestive proteins involved in the reuptake of trehalose in the Malpighian tubules of insects [68]. Trehalose is the main sugar nutrient in most insects and also plays a role in protection against heat, cold, and desiccation (reviewed in [69,70]). In addition to general digestion, we identified UDP-glucuronosyltransferases (*UGT*) that can facilitate detoxification [71]. UGTs do have other functions that may not be involved in predatory behavior, such as olfaction, cuticle formation, and pigmentation [72,73,74]. 

Adding to the morphological genes putatively involved in predatory behavior, we also identified takeout proteins and takeout-like proteins, which have been found to be involved in male courtship behavior of *Drosophila* [75]. Specifically, a knockout experiment showed reduced probability that male *Drosophila* would court females past the initial steps of orientation and following. Takeout proteins could be under positive selection due to either predatory behavior, because of aggressive mimicry, or just general differences in courtship behavior. 

#### 3.2.3. Positively Selected Genes Common to All Comparisons 

Found in both of the control comparisons, ubiquinone biosynthesis o-methyltransferase functions in the ubiquinone biosynthesis pathway [76]. Ubiquinone is found in all eukaryotes and functions as part of the respiratory electron transport system within the inner mitochondrial membrane. Additionally, ubiquinol, a reduced form of ubiquinone is found to help prevent oxidative damage [76,77,78]. This may be a major difference between *Photuris* and *Photinus*, because it was found in only the comparisons that compared the two genera.

Zinc finger proteins were found to be under divergent selection in all comparisons. These proteins have diverse functions, including involvement with *lola* function [56]. Zinc finger proteins are one of the most widely abundant transcription factors for DNA-binding in eukaryotes (reviewed in [79]). In both of the *Photinus*-*Photuris* comparisons, two gene families with homology to zinc finger proteins were identified as under positive selection. However, each of those families is composed of sequences from only one species in the comparison. This suggests that these proteins are under positive selection for functional divergence of gene duplicates within-species, as observed in other metazoans [80]. Our pipeline did not cluster these proteins from *Photuris* and *Photinus* together, possibly due to extensive divergence. The ubiquity of zinc finger protein identification by our pipeline and their known diversity across animal taxa suggests that these proteins may be diverging among all species of fireflies.

### 3.3. Implications for Evolution of Predation in Fireflies

Our results, leveraging the power of high-throughput sequencing and comparative analysis in non-model organisms, provide a first intriguing glimpse into genes potentially involved in the *femme fatale* predatory phenotype. Importantly, these results generate specific hypotheses for genes potentially related to predation in fireflies that can be tested in future studies. It is possible that future work will identify additional genes implicated in this behavior. Here, we were limited to analysis of existing male head-specific datasets for both *Photuris* species. Due to existing evidence that males can prey upon other fireflies and may do so in the wild, we expect that genes involved in predation are included in our analysis [23]. However, any genes expressed only in *Photuris* females during predatory behavior may be missing from the analysis. Additionally, any genes involved in adult predatory behavior that are expressed in tissues other than the head, such as the abdominal ganglia, may be missing. Future RNA-seq studies to investigate the expression of these genes in relation to the predatory phenotype across species, tissues, life stages, and sexes will provide more complete transcriptome assemblies and additional support for the roles of these candidate genes in predation. Furthermore, functional characterization of candidate proteins will provide functional confirmation of their roles in predation. Finally, the genes we identified could have implications for other predatory/feeding behaviors and behavioral switches across insects, such as feeding behavior in mosquitos [81] and temporal polyethism in honey bee nest behavior [82], helping us to untangle the genetic intricacies of phenotypic variation in complex behaviors across insects.

## Figures and Tables

**Table 1:** Assembly statistics for three firefly species. Statistics compiled from Trinity assembly assessment output (script name/flags). N genes = number of unique genes in assembly, N transcripts = number of unique transcripts (including isoforms) in assembly. GC (%) = GC content percentage in assembly. Median length (bp) = median length of longest isoforms per gene. Mean length (bp) = mean length of longest isoforms per gene. N bases = number of total bases in assembly based on longest isoforms per gene only. N50 = N50 statistic based on longest isoforms per gene only. Assembly statistics for all transcripts, not just longest isoforms, are given in (Supplemental Table S2).

Species	N Genes	N Transcripts	GC (%)	Median Length (bp)	Mean Length (bp)	N Bases	N50
*Photuris frontalis*	40,547	58,028	34.65	405	866.46	35,132,369	1693
*Photuris* sp.	38,303	56,626	34.43	402	901.44	34,527,893	1820
*Photinus pyralis*	130,648	188,474	38.96	346	634.52	82,898,270	902

**Table 2:** Benchmarking Universal Single-Copy Orthologs (BUSCO) assembly scores. C = Complete BUSCOs. S = Complete and single-copy BUSCOs. D = Complete and duplicated BUSCOs. F = Fragmented BUSCOs. M = Missing BUSCOs.

Species	C (%)	S (%)	D (%)	F (%)	M (%)
*Photuris frontalis*	90.9%	62.2%	28.7%	4.9%	4.2%
*Photuris* sp.	91.3%	59.6%	31.7%	4.4%	4.3%
*Photinus pyralis*	93.5%	46.3%	47.2%	3.9%	2.6%

**Table 3:** FUSTr results. Comparison = the two species FUSTr compared in its analysis. N input transcripts = number of transcripts input into FUSTr. N isoforms disregarded = number of isoforms removed from analysis. N transcripts used = number of transcripts used by FUSTr. N families = number of gene families found in each comparison. N families with > 15 sequences = number of gene families with greater than 15 sequences. N families with β > α = number of gene families found to be under strong positive selection.

Comparison	N input Transcripts	N Isoforms Disregarded	N Transcripts Used	N Families	N Families with > 15 Sequences	N Families with β > α
*Pt.* sp. vs. *Pt. frontalis*	114,654	29,237	46,371	36,387	44	9
*Pt.* sp. vs. *Pn. pyralis*	245,100	49,882	71,700	62,055	49	12
*Pt. frontalis* vs. *Pn. pyralis*	246,502	49,025	74,039	64,354	48	8

## Supplementary Materials

The following are available online at https://www.mdpi.com/2073-4425/11/6/627/s1, *Pn. pyralis* head tissue analysis, Table S1: Raw read data, Table S2: Trinity assembly statistics based on all transcript contigs, Table S3: Previously published BUSCO scores. Table S4: Putative functions of genes under positive selection in a *Photuris* sp. vs. *Photuris frontalis* comparison. Table S5: Putative functions of genes under positive selection in a *Photuris frontalis* vs. *Photinus pyralis* comparison. Table S6: Putative functions of genes under positive selection in a *Photuris* sp. vs. *Photinus pyralis* comparison, Table S7: Assembly statistics for *Photinus pyralis* head tissue assembly based on all transcript contigs, Table S8: Assembly statistics for *Photinus pyralis* head tissue assembly based on only the longest isoform per gene, Table S9: BUSCO assembly scores, Table S10: FUSTr Results, Table S11: Homologs of genes under positive selection in a *Photuris frontalis* and *Photinus pyralis* head tissue comparison identified by BLAST, Table S12: Homologs of genes under positive selection in a *Photuris* sp. and *Photinus pyralis* head tissue comparison identified by BLAST. Additional supplementary data, including transcriptome assemblies, FUSTr output, and BLAST results are available via FigShare: https://figshare.com/s/38b4053f456ec57b1e46.

## References

[1] Thomas A.L., Davis S.M., Dierick H.A. (2015). Of fighting flies, mice, and men: Are some of the molecular and neuronal mechanisms of aggression universal in the animal kingdom?. PLoS Genet..

[2] Konopka R.J., Benzer S. (1971). Clock mutants of *Drosophila melanogaster*. Proc. Natl. Acad. Sci. USA.

[3] Hall J.C. (1978). Courtship among males due to a male-sterile mutation in *Drosophila melanogaster*. Behav. Genet..

[4] York R.A. (2018). Assessing the genetic landscape of animal behavior. Genetics.

[5] Zhong H., Zhang H., Tang Z., Guo Z., Yan J., Xiao J., Luo Y., Zhou Y. (2018). Evidence for natural selection of immune genes from *Parachromis managuensis* by transcriptome sequencing. Biotechnol. Biotech. Eq..

[6] Heras J., Aguilar A. (2019). Comparative transcriptomics reveals patterns of adaptive evolution associated with depth and age within marine rockfishes (Sebastes). J. Hered..

[7] Zhang J., Xie P., Lascoux M., Meagher T.R., Liu J. (2013). Rapidly evolving genes and stress adaptation of two desert poplars, *Populus euphratica* and *P. pruinosa*. PLoS ONE.

[8] Murrell B., Moola S., Mabona A., Weighill T., Sheward D., Kosakovsky Pond S.L., Scheffler K. (2013). FUBAR: A fast, unconstrained bayesian approximation for inferring selection. Mol. Biol. Evol..

[9] Balakrishnan C.N., Chapus C., Brewer M.S., Clayton D.F. (2013). Brain transcriptome of the violet-eared waxbill *Uraeginthus granatina* and recent evolution in the songbird genome. Open Biol..

[10] Barreto F.S., Moy G.W., Burton R.S. (2011). Interpopulation patterns of divergence and selection across the transcriptome of the copepod *Tigriopus californicus*. Mol. Ecol..

[11] Brewer M.S., Carter R.A., Croucher P.J.P., Gillespie R.G. (2015). Shifting habitats, morphology, and selective pressures: Developmental polyphenism in an adaptive radiation of Hawaiian spiders. Evolution.

[12] Lewis S.M., Cratsley C.K. (2008). Flash signal evolution, mate choice, and predation in fireflies. Annu. Rev. Entomol..

[13] Sander S.E., Hall D.W. (2015). Variation in opsin genes correlates with signalling ecology in North American fireflies. Mol. Ecol..

[14] Amaral D.T., Silva J.R., Viviani V.R. (2017). Transcriptomes from the photogenic and non-photogenetic tissues and life stages of the *Aspisoma lineatum* firefly (Coleoptera: Lampyridae): Implications for the evolutionary origins of bioluminescence and its associated light organs. Gene Rep..

[15] Fallon T.R., Lower S.E., Chang C.-H., Bessho-Uehara M., Martin G.J., Bewick A.J., Behringer M., Debat H.J., Wong I., Day J.C. (2018). Firefly genomes illuminate parallel origins of bioluminescence in beetles. eLife.

[16] Fu X., Li J., Tian Y., Quan W., Zhang S., Liu Q., Liang F., Zhu X., Zhang L., Wang D. (2017). Long-read sequence assembly of the firefly *Pyrocoelia pectoralis* genome. Gigascience.

[17] Lloyd J.E. (1966). Studies on the Flash Communication System in Photinus Fireflies.

[18] Stanger-Hall K.F., Lloyd J.E. (2015). Flash signal evolution in *Photinus* fireflies: Character displacement and signal exploitation in a visual communication system. Evolution.

[19] Faust L.F. (2017). Fireflies, Glow-Worms, and Lightning Bugs: Identification and Natural History of the Fireflies of the Eastern and Central United States and Canada.

[20] Eisner T., Goetz M.A., Hill D.E., Smedley S.R., Meinwald J. (1997). Firefly “femmes fatales” acquire defensive steroids (lucibufagins) from their firefly prey. Proc. Natl. Acad. Sci. USA.

[21] Deyrup S.T., Risteen R.G., Tonyai K.K., Farrar M.A., D’Antonio B.E., Ahmed Z.B., Christofel B.T., Howells N.R., Smedley S.R. (2017). Escape into winter: Does a phenological shift by *Ellychnia corrusca* (Winter Firefly) shield it from a specialist predator (*Photuris*)?. Northeast. Nat..

[22] Faust L., De Cock R., Lewis S. (2012). Thieves in the night: Kleptoparasitism by fireflies in the genus *Photuris* Dejean (Coleoptera: Lampyridae). Coleopts Bull..

[23] Barber H.S. (1951). North American fireflies of the genus *Photuris*. Smithson. Misc. Collect..

[24] Lloyd J.E., Wing S.R. (1983). Nocturnal aerial predation of fireflies by light-seeking fireflies. Science.

[25] Lloyd J.E. (1965). Aggressive mimicry in *Photuris*: Firefly femmes fatales. Science.

[26] Zorn L.P., Carlson A.D. (1978). Effect of mating on response of female *Photuris* firefly. Anim. Behav..

[27] Heckscher C.M. (2013). *Photuris mysticalampas* (Coleoptera: Lampyridae): A new firefly from peatland floodplain forests of the Delmarva peninsula. Entomol. News.

[28] Crespi B.J. (2017). Shared sociogenetic basis of honey bee behavior and human risk for autism. Proc. Natl. Acad. Sci. USA.

[29] McLean M., Buck J., Hanson F.E. (1972). Culture and larval behavior of Photurid fireflies. Am. Midl. Nat..

[30] SRA Toolkit Development Team SRA Toolkit. http://ncbi.github.io/sra-tools/.

[31] Fallon T.R., Li F.-S., Vicent M.A., Weng J.-K. (2016). Sulfoluciferin is biosynthesized by a specialized luciferin sulfotransferase in fireflies. Biochemistry.

[32] Al-Wathiqui N., Fallon T.R., South A., Weng J.-K., Lewis S.M. (2016). Molecular characterization of firefly nuptial gifts: A multi-omics approach sheds light on postcopulatory sexual selection. Sci. Rep..

[33] Grabherr M.G., Haas B.J., Yassour M., Levin J.Z., Thompson D.A., Amit I., Adiconis X., Fan L., Raychowdhury R., Zeng Q. (2011). Full-length transcriptome assembly from RNA-Seq data without a reference genome. Nat. Biotech..

[34] Macmanes M.D. (2014). On the optimal trimming of high-throughput mRNA sequence data. Front. Genet..

[35] Simão F.A., Waterhouse R.M., Ioannidis P., Kriventseva E.V., Zdobnov E.M. (2015). BUSCO: Assessing genome assembly and annotation completeness with single-copy orthologs. Bioinformatics.

[36] Cole T.J., Brewer M.S. (2018). FUSTr: A tool to find gene families under selection in transcriptomes. PeerJ.

[37] Köster J., Rahmann S. (2018). Snakemake-a scalable bioinformatics workflow engine. Bioinformatics.

[38] Haas B.J., Papanicolaou A., Yassour M., Grabherr M., Blood P.D., Bowden J., Couger M.B., Eccles D., Li B., Lieber M. (2013). *De novo* transcript sequence reconstruction from RNA-seq using the Trinity platform for reference generation and analysis. Nat. Protoc..

[39] Altschul S.F., Gish W., Miller W., Myers E.W., Lipman D.J. (1990). Basic local alignment search tool. J. Mol. Biol..

[40] Miele V., Penel S., Duret L. (2011). Ultra-fast sequence clustering from similarity networks with SiLiX. BMC Bioinform..

[41] Yang Z. (2007). PAML 4: Phylogenetic analysis by maximum likelihood. Mol. Biol. Evol..

[42] Hofmann L., Tsybovsky Y., Alexander N.S., Babino D., Leung N.Y., Montell C., Banerjee S., von Lintig J., Palczewski K. (2016). Structural Insights into the *Drosophila melanogaster* retinol dehydrogenase, a member of the short-chain dehydrogenase/reductase family. Biochemistry.

[43] Sumayao R., Newsholme P., McMorrow T. (2018). The role of cystinosin in the intermediary thiol metabolism and redox homeostasis in kidney proximal tubular cells. Antioxid. Basel.

[44] Shi H., Pei L., Gu S., Zhu S., Wang Y., Zhang Y., Li B. (2012). Glutathione S-transferase (GST) genes in the red flour beetle, *Tribolium castaneum*, and comparative analysis with five additional insects. Genomics.

[45] González A., Hare J.F., Eisner T. (1999). Chemical egg defense in Photuris firefly “femmes fatales”. Chemoecology.

[46] Mayoral J.G., Nouzova M., Navare A., Noriega F.G. (2009). NADP+-dependent farnesol dehydrogenase, a corpora allata enzyme involved in juvenile hormone synthesis. Proc. Natl. Acad. Sci. USA.

[47] Nijhout F.H. (1998). Insect Hormones.

[48] Li J., Choo Y.M., Lee K.S., Je Y.H., Woo S.D., Kim I., Sohn H.D., Jin B.R. (2005). A serine protease gene from the firefly, *Pyrocoelia rufa*: Gene structure, expression, and enzyme activity. Biotechnol. Lett..

[49] Luo W., Wang Y., Reiser G. (2007). Protease-activated receptors in the brain: Receptor expression, activation, and functions in neurodegeneration and neuroprotection. Brain Res. Rev..

[50] Wang Y., Luo W., Reiser G. (2008). Trypsin and trypsin-like proteases in the brain: Proteolysis and cellular functions. Cell Mol. Life Sci..

[51] Turgeon V.L., Houenou L.J. (1997). The role of thrombin-like (serine) proteases in the development, plasticity and pathology of the nervous system. Brain Res. Rev..

[52] Davies B.J., Pickard B.S., Steel M., Morris R.G.M., Lathe R. (1998). Serine proteases in rodent hippocampus. J. Biol. Chem..

[53] Gingrich M.B., Traynelis S.F. (2000). Serine proteases and brain damage—Is there a link?. Trends Neurosci..

[54] Almonte A.G., Sweatt J.D. (2011). Serine proteases, serine protease inhibitors, and protease-activated receptors: Roles in synaptic function and behavior. Brain Res..

[55] Giniger E., Tietje K., Jan L.Y., Jan Y.N. (1994). *Lola* encodes a putative transcription factor required for axon growth and guidance in *Drosophila*. Development.

[56] Sato K., Ito H., Yokoyama A., Toba G., Yamamoto D. (2019). Partial proteasomal degradation of *Lola* triggers the male-to-female switch of a dimorphic courtship circuit. Nat. Commun..

[57] Lin J., Patel S.R., Cheng X., Cho E.A., Levitan I., Ullenbruch M., Phan S.H., Park J.M., Dressler G.R. (2005). Kielin/chordin-like protein, a novel enhancer of BMP signaling, attenuates renal fibrotic disease. Nat. Med..

[58] Li H.-Y., Lin X.-W., Geng S.-L., Xu W.-H. (2018). TGF-β and BMP signals regulate insect diapause through Smad1-POU-TFAM pathway. Biochim. Biophys. Acta Mol. Cell Res..

[59] Meekins D.A., Kanost M.R., Michel K. (2017). Serpins in arthropod biology. Semin. Cell Dev. Biol..

[60] Cohen S.S. (1998). A Guide to the Polyamines.

[61] Wallace H.M., Fraser A.V., Hughes A. (2003). A perspective of polyamine metabolism. Biochem. J..

[62] Zhou J.-J. (2010). Odorant-binding proteins in insects. Vitam. Horm..

[63] Shinoda T., Itoyama K. (2003). Juvenile hormone acid methyltransferase: A key regulatory enzyme for insect metamorphosis. Proc. Natl. Acad. Sci. USA.

[64] Cavener D.R. (1992). GMC oxidoreductases: A newly defined family of homologous proteins with diverse catalytic activities. J. Mol. Biol..

[65] Bak T.-G. (1967). Studies on glucose dehydrogenase of aspergillus oryzae. Biochim. Biophys. Acta Enzymol..

[66] Murtha M.T., Cavener D.R. (1989). Ecdysteroid regulation of glucose dehydrogenase and alcohol dehydrogenase gene expression in *Drosophila melanogaster*. Dev. Biol..

[67] Iida K., Cavener D.R. (2004). Glucose dehydrogenase is required for normal sperm storage and utilization in female *Drosophila melanogaster*. J. Exp. Biol..

[68] Kikuta S., Hagiwara-Komoda Y., Noda H., Kikawada T. (2012). A novel member of the trehalose transporter family functions as an h(+)-dependent trehalose transporter in the reabsorption of trehalose in malpighian tubules. Front. Physiol..

[69] Crowe J.H., Carpenter J.F., Crowe L.M. (1998). The role of vitrification in anhydrobiosis. Annu. Rev. Physiol..

[70] Arrese E.L., Soulages J.L. (2010). Insect fat body: Energy, metabolism, and regulation. Annu. Rev. Entomol..

[71] Meech R., Mackenzie P.I. (1997). Structure and function of uridine diphosphate glucuronosyltransferases. Clin. Exp. Pharmacol. Physiol..

[72] Lazard D., Zupko K., Poria Y., Nef P., Lazarovits J., Horn S., Khen M., Lancet D. (1991). Odorant signal termination by olfactory UDP glucuronosyl transferase. Nature.

[73] Hopkins T.L., Kramer K.J. (1992). Insect cuticle sclerotization. Annu. Rev. Entomol..

[74] Wiesen B., Krug E., Fiedler K., Wray V., Proksch P. (1994). Sequestration of host-plant-derived flavonoids by lycaenid butterfly *Polyommatus icarus*. J. Chem. Ecol..

[75] Dauwalder B., Tsujimoto S., Moss J., Mattox W. (2002). The *Drosophila* takeout gene is regulated by the somatic sex-determination pathway and affects male courtship behavior. Genes Dev..

[76] Li H., Young B.J., Wang H., Schooley D.A. (1998). The structure of ubiquinones isolated from developing embryos of *Manduca sexta*. Insect Biochem. Molec..

[77] Beyer R.E. (1992). An analysis of the role of coenzyme Q in free radical generation and as an antioxidant. Biochem. Cell Biol..

[78] Ernster L., Dallner G. (1995). Biochemical, physiological and medical aspects of ubiquinone function. Biochim. Biophys. Acta.

[79] Laity J.H., Lee B.M., Wright P.E. (2001). Zinc finger proteins: New insights into structural and functional diversity. Curr. Opin. Struct. Biol..

[80] Emerson R.O., Thomas J.H. (2009). Adaptive evolution in zinc finger transcription factors. PLoS Genet..

[81] Beier J.C. (1996). Frequent blood-feeding and restrictive sugar-feeding behavior enhance the malaria vector potential of *Anopheles gambiae* s.l. and *An. funestus* (Diptera: Culicidae) in western Kenya. J. Med. Entomol..

[82] Robinson G.E. (1992). Regulation of division of labor in insect societies. Annu. Rev. Entomol..

