# Supplementary material for: Comparative Transcriptomics Reveals Gene Families Associated with Predatory Behavior in Photuris femme fatale Fireflies
Source: Genes (Basel). 2020 Jun 7;11(6):627. doi: 10.3390/genes11060627 (PMC7348864; doi:10.3390/genes11060627)
Supplement: Supplementary file 1 [file genes-11-00627-s001.pdf]

Supplemental Text 1: *Pn. pyralis* head tissue analysis:

Materials and Methods:

To investigate the effect of using multiple individuals for the *Photinus pyralis* assembly on our results, we repeated the assembly and analyses using RNAseq data from a single *Pn. pyralis* male head (SRR2103848, Sander and Hall 2015).

Results and Discussion:

2.8 Gb of sequence data were used to assemble the *Pn. pyralis* transcriptome. The resulting assembly has a similar number of transcripts to the *Photuris* head-only transcriptome assemblies (Supplemental Table 7 and Table 1). The BUSCO score is also slightly higher than the previous assembly (Supplemental Table 9 and Table 2).

Species comparisons identify gene families under selection

The FUSTr analysis found 12 total gene families with evidence for positive selection in the control comparisons (Supplemental Table 10). All of these gene families were found in the original analysis. We note the similarities and the differences between the original and new analysis for each comparison below:

Similarities and differences in the Photinus pyralis (head tissue) and Photuris frontalis (non-predatory) comparison.

In this new *Pn. pyralis* and *Pt. frontalis* comparison, we identified three of the same gene families found in the previous analysis: kielin/chordin like protein and two zinc finger protein homologs (Supplemental Table 11 and Figure 2). All other gene families in the original analysis were not found in these results (4-coumarate--CoA ligase 1-like, antichymotrypsin-2, ubiquinone biosynthesis O-methyltransferase, uncharacterized protein, and a family with no BLAST hits; Supplemental Table 11 and Figure 2).

Two gene families that were found in other comparisons in the original results were found as positively selected (Supplemental Table 11 and Figure 2). Farnesol dehydrogenase was originally found in the *Pt. sp.* (predatory) and *Pt. frontalis* (non-predatory) comparison. Farnesol dehydrogenase is involved in juvenile hormone (JH) production (Mayoral et al. 2009). We hypothesize that the function of the JH may be relevant to predatory behavior due to its ability to be a gonadotropin. Because this analysis is composed of only non-predatory species, it is possible that farnesol dehydrogenase is not involved in predatory behavior. Additionally, we identified UDP-glucuronosyltransferase as positively selected in this comparison, however, it was originally found in only the *Pt. sp.* (predatory) and *Pn. pyralis* (control) comparison. UDP-glucuronosyltransferase can facilitate detoxification, and it was for this reason that we thought it could be involved in predatory behavior (Meech and Mackenzie 1997). However, it also functions in olfaction, cuticle formation, and pigmentation (Lazard et al. 1991, Hopkins and Kramer 1992, and Wiesen et al. 1994). These functions are more likely due to the non-predatory nature of this comparison.

Similarities and differences in the Photinus pyralis (head tissue) and Photuris sp. (predatory) comparison.

In this new *Pn. pyralis* and *Pt. sp.* comparison, we identified five of the same gene families found in the previous analysis: UDP-glucuronosyltransferase, spermine oxidase, uncharacterized protein, and two zinc finger protein homologs (Supplemental Table 12 and Figure 2). All other gene families in the original analysis were not found in these results (facilitated trehalose transporter Tret1-like, glucose dehydrogenase, juvenile hormone acid O-methyltransferase, odorant binding protein, takeout protein, and ubiquinone biosynthesis O-methyltransferase.

Two gene families that were originally found in other comparisons in the original results were found as positively selected (Supplemental Table 12 and Figure 2). Farnesol dehydrogenase was also found in this comparison, therefore it was found in all comparisons. This shows that it may not be involved in predatory behavior. Antichymotrypsin was originally found in the *Pn. pyralis* and *Pt. frontalis* comparison. Now, it is found in the *Pn. pyralis* (head tissue) and *Pt. sp.* comparison, and not in the *Pn. pyralis* (head tissue) and *Pt. frontalis* comparison. Antichymotrypsin is a member of the serpin family, which have been found to be involved in innate immunity

(Meekins et al. 2017). Because antichymotrypsin was originally found in the non-predatory vs. control comparison, we would expect it to not be involved in predatory behavior.

Gene families not identified in the new analysis.

There was a total of 11 gene families not found in the new analysis across comparisons (Supplemental Tables 11-12, Figure 2). This is likely due to the smaller number of tissues, thus, and thus, lower number of transcripts in the new *Pn. pyralis* transcriptome assembly.

Supplemental Table 1: Raw read data: SRA Number = Short read archive number for sample. Tissue = tissue the sample was extracted from. Accession = NCBI accession number. Length = read length. N Spots = number of spots. N Bases = number of basepairs.

Species	SRA Number	Tissue	Accession	Length (bp)	N Spots	N Bases (G)
Photinus pyralis	SRR2103848	male head	PRJNA289908	100	14246268	2.8
Photinus pyralis	SRR2103849	male light organ	PRJNA289908	100	11816288	2.4
Photinus pyralis	SRR2103867	larval light organ	PRJNA289908	100	13007015	2.6
Photinus pyralis	SRR3521424	posterior abdomen	PRJNA321737	126	20140685	4.2
Photinus pyralis	SRR3883756	FatBody	PRJNA328865	100	11045965	2.2
Photinus pyralis	SRR3883757	FatBody	PRJNA328865	100	8696048	1.7
Photinus pyralis	SRR3883758	Male other reproductive accessory glands	PRJNA328865	100	11037319	2.2
Photinus pyralis	SRR3883759	Male other reproductive accessory glands post mating	PRJNA328865	100	7455988	1.5
Photinus pyralis	SRR3883760	Female spermatophore digesting glands and Bursa	PRJNA328865	100	9439392	1.9
Photinus pyralis	SRR3883761	Male reproductive spiral glands post mating	PRJNA328865	100	9223507	1.8
Photinus pyralis	SRR3883762	Female spermatheca	PRJNA328865	100	12232743	2.4
Photinus pyralis	SRR3883763	Male reproductive spiral gland	PRJNA328865	100	14616067	2.9
Photinus pyralis	SRR3883764	Male reproductive spiral gland	PRJNA328865	100	7932593	1.6
Photinus	SRR388	Male reproductive spiral gland	PRJNA3	100	6457676	1.3

pyralis	3765		28865			
Photinus pyralis	SRR3883766	FatBody	PRJNA328865	100	9025721	1.8
Photinus pyralis	SRR3883767	FatBody post mating	PRJNA328865	100	12981252	2.6
Photinus pyralis	SRR3883768	Female thorax	PRJNA328865	100	8574669	1.7
Photinus pyralis	SRR3883769	Male throax	PRJNA328865	100	11164875	1.2
Photinus pyralis	SRR3883770	Male throax	PRJNA328865	100	7176451	1.4
Photinus pyralis	SRR3883771	Male throax	PRJNA328865	100	7400466	1.5
Photinus pyralis	SRR3883772	Male other reproductive accessory glands	PRJNA328865	100	8461346	1.7
Photinus pyralis	SRR3883773	Male other reproductive accessory glands	PRJNA328865	100	10194748	2
Photinus pyralis	SRR6345445	larvae	PRJNA378805	150	31282614	9.4
Photinus pyralis	SRR6345446	adult male dissected photophore	PRJNA378805	100	15475496	3.1
Photinus pyralis	SRR6345447	eggs	PRJNA378805	150	44551762	13.4
Photinus pyralis	SRR6345449	adult female whole body	PRJNA378805	150	31480011	9.4
Photinus pyralis	SRR6345452	adult male whole body	PRJNA378805	150	31194573	9.4
Photinus pyralis	SRR6345453	adult male dissected photophore	PRJNA378805	100	9281220	1.9
Photinus pyralis	SRR6345454	adult male dissected photophore	PRJNA378805	100	9058347	1.8
Photinus pyralis	SRR7345580	3 pooled 4th instar larvae dissected photophore	PRJNA378805	100	43423999	8.7
Photuris frontalis	SRR2104391	male head	PRJNA289908	100	11373006	2.3
Photuris sp.	SRR2104392	male head	PRJNA289908	100	12359044	2.5

Supplemental Table 2: Trinity assembly statistics based on all transcript contigs.

Species	Median contig length	Average contig length	Total assembled bases	N50
Photinus pyralis	404	932.84	175816477	2030
Photuris sp.	591	1295.24	73344107	2602
Photuris frontalis	554	1197	69459423	2353

Supplemental Table 3: Previously published assembly BUSCO scores. (Sander and Hall 2015; Fallon et al. 2018)

Species	Complete BUSCOs	Complete and single -copy BUSCOs	Complete and duplicated BUSCOs	Fragmented BUSCOs	Missing BUSCOs	Total BUSCO groups searched
Photuris frontalis	88.0%	65.8%	22.2%	6.4%	5.6%	2442
Photuris sp.	88.8%	67.2%	21.6%	5.9%	5.3%	2442
Photinus pyralis	95.2%	60.4%	34.8%	2.5%	2.3%	2442

Supplemental Table 4. Homologs of genes under positive selection in *Photuris sp.* vs. *Photuris frontalis* comparison identified by BLAST. Gene family = arbitrarily assigned gene family number from FUSTr. N genes = number of genes in the gene family. N sites under selection = amino acid sites under positive selection in the consensus sequence. N BLAST hits = number of significant (evalue cutoff of $1e^{-5}$) BLAST hits within that gene family. N genes with BLAST hits = number of genes in the gene family that had BLAST hits. N genes *Photuris sp.* = number of genes in the family that are from *Photuris sp.* N genes *Photuris frontalis* - number of genes in the family that are from *Photuris frontalis*. BLAST homolog = the homolog that a majority of the gene family had a BLAST hit for.

**Gene family**	**N genes**	**N sites under positive selection**	**N BLAST hits**	**N genes with BLAST hits**	**N genes *Pt. sp.***	**N genes *Pt. frontalis***	**BLAST homolog**
553	18	1	737	18	7	11	Retinol dehydrogenase
987	15	1	448	15	8	7	Cystinosin homolog
470	38	1	912	38	20	18	Glutathione S-transferase
2079	40	3	1248	40	21	19	Farnesol dehydrogenase
964	24	3	800	24	13	11	Serine protease
381	20	1	1075	20	9	11	Transmembrane protease serine
86	19	3	260	19	7	12	Longitudinals lacking protein (*lola*)

306	156	4	319	26	68	88	Zinc finger protein
1478	26	1	362	26	11	15	Uncharacterized protein
Total	356		6161	226	164	192	

Supplemental Table 5. Homologs of genes under positive selection in *Photuris frontalis* vs. *Photinus pyralis* comparison identified by BLAST. Gene family = arbitrarily assigned gene family number from FUSTr. N genes = number of genes in the gene family. N sites under selection = amino acid sites under positive selection in the consensus sequence. N BLAST hits = number of significant (evalue cutoff of $1e^{-5}$) BLAST hits within that gene family. N genes with BLAST hits = number of genes in the gene family that had BLAST hits. N genes *Photuris frontalis.* = number of genes in the family that are from *Photuris frontalis.* N genes *Photinus pyralis* - number of genes in the family that are from *Photinus pyralis*. BLAST homolog = the homolog that a majority of the gene family had a BLAST hit for.

**Gene family**	**N genes**	**N sites under positive selection**	**N BLAST hits**	**N genes with BLAST hits**	**N genes *Pt. frontalis***	**N genes *Pn. pyralis***	**BLAST homolog**
881	21	1	106	13	16	5	Kielin/chordin-like protein/protease inhibitor
238	19	1	504	19	7	12	Antichymotrypsin-2
30	21	2	840	22	10	11	4-coumarate--CoA ligase 1-like

383	28	1	560	28	15	13	Ubiquinone biosynthesis O-methyltransferase, mitochondrial
261	69	2	229	16	69	0	Zinc-finger protein
5627	121	2	194	14	0	121	Zinc-finger protein
1255	38	1	2752	14	4	34	Uncharacterized protein
218	20	1	0	0	3	17	No BLAST hits
Total	337		5185	149	124	213	

Supplemental Table 6. Homologs of genes under positive selection in *Photuris sp.* vs. *Photinus pyralis* comparison identified by BLAST. Gene family = arbitrarily assigned gene family number from FUSTr. N genes = number of genes in the gene family. N sites under selection = amino acid sites under positive selection in the consensus sequence. N BLAST hits = number of significant (evalue cutoff of $1e^{-5}$) BLAST hits within that gene family. N genes with BLAST hits = number of genes in the gene family that had BLAST hits. N genes *Photuris sp.* = number of genes in the family that are from *Photuris sp.* N genes *Photinus pyralis* - number of genes in the family that are from *Photinus pyralis.* BLAST homolog = the homolog that a majority of the gene family had a BLAST hit for.

**Gene family**	**N genes**	**N sites under positive selection**	**N BLAST hits**	**N genes with BLAST hits**	**N genes *Pt. sp.***	**N genes *Pn. pyralis***	**BLAST homolog**

1810	17	1	865	17	6	11	Spermine oxidase
157	15	1	5	1	1	14	Odorant binding protein
517	25	1	343	25	6	19	Juvenile hormone acid O-methyltransferase
365	35	1	2337	34	13	22	Glucose dehydrogenase
759	21	1	822	21	10	11	Facilitated trehalose transporter Tret1-like
347	52	2	2510	52	23	29	UDP-glucuronosyltransferase
1231	15	2	242	15	3	12	Protein takeout
686	25	1	503	25	12	13	Ubiquinone biosynthesis O-methyltransferase
21	121	1	194	17	0	121	Zinc finger protein

5939	49	1	74	7	49	0	Zinc finger protein
1017	20	1	1040	20	7	13	Uncharacterized protein
15	28	1	354	28	11	17	Uncharacterized protein
Total	423		8935	255	141	282	

Supplemental Table 7. Assembly statistics for *Photinus pyralis* head tissue assembly based on all transcript contigs. Statistics compiled from Trinity assembly assessment output (script name/flags). N genes = Number of unique genes in assembly, N transcripts = number of unique transcripts (including isoforms) in assembly. GC (%) = GC content percentage in assembly. Median length (bp) = median length. Mean length (bp) = mean length. N bases = Number of total bases in assembly. N50 = N50 statistic.

Species	N genes	N transcripts	GC (%)	Median length (bp)	Mean length (bp)	N bases	N50
Photinus pyralis (head)	32017	49482	40.38	730	1473.50	72911734	2855

Supplemental Table 8. Assembly statistics for *Photinus pyralis* head tissue assembly based on only the longest isoform per gene. Median length (bp) = median length of longest isoforms per gene. Mean length (bp) = mean length of longest isoforms per gene. N bases = Number of total bases in assembly. N50 = N50 statistic.

Species	Median length (bp)	Mean length (bp)	N bases	N50
Photinus pyralis (head)	427	1014.25	32473248	2094

Supplemental Table 9. BUSCO assembly scores. C = Complete BUSCOs. S = Complete and single-copy BUSCOs. D = Complete and duplicated BUSCOs. F = Fragmented BUSCOs. M = Missing BUSCOs.

Species	C (%)	S (%)	D (%)	F (%)	M (%)

Photinus pyralis (head)	94.1%	58.9%	35.2%	2.7%	3.2%

Supplemental Table 10. FUSTr results. Comparison = the two species FUSTr compared in its analysis. N input transcripts = number of transcripts input into FUSTr. N isoforms disregarded = number of isoforms removed from analysis. N transcripts used = number of transcripts used by FUSTr. N families = number of gene families found in each comparison. N families with > 15 sequences = number of gene families with greater than 15 sequences. N families with β > α = number of gene families found to be under strong positive selection.

Comparison	N input transcripts	N Isoforms disregarded	N Transcripts used	N Families	N Families with > 15 sequences	N Families with β > α
Pt. frontalis and *Pn. pyralis*	107510	28364	43471	34576	39	5
Pt. sp. and *Pn. pyralis*	106108	29221	41132	32256	37	7

Supplemental Table 11. Homologs of genes under positive selection in *Photuris frontalis* and *Photinus pyralis* head tissue comparison identified by BLAST. Gene family = arbitrarily assigned gene family number from FUSTr. N genes = number of genes in the gene family. N sites under selection = amino acid sites under positive selection in the consensus sequence. N BLAST hits = number of significant (evalue cutoff of 1e-5) BLAST hits within that gene family. N genes with BLAST hits = number of genes in the gene family that had BLAST hits. BLAST homolog = the homolog that a majority of the gene family had a BLAST hit for.

Gene Family	N Genes	N sites under selection	N BLAST hits	N Genes with BLAST hits	BLAST homolog
5955	76	2	109	15	zinc finger protein
1820	40	1	1236	38	Farnesol dehydrogenase
939	23	1	99	12	kielin/chordin like protein
280	48	1	2314	47	UDP-glucuronosyltransferase
266	68	2	215	16	zinc finger protein
Total	255	7	3973	128	

Supplemental Table 12. Homologs of genes under positive selection in *Photuris sp.* and *Photinus pyralis* head tissue comparison identified by BLAST. Gene family = arbitrarily assigned gene family number from FUSTr. N genes = number of genes in the gene family. N sites under selection = amino acid sites under positive selection in the consensus sequence. N BLAST hits = number of significant (evalue cutoff of 1e-5) BLAST hits within that gene family. N genes with BLAST hits = number of genes in the gene family that had BLAST hits. BLAST homolog = the homolog that a majority of the gene family had a BLAST hit for.

Gene Family	N Genes	N sites under selection	N BLAST hits	N Genes with BLAST hits	BLAST homolog
19	43	1	1348	43	farnesol dehydrogenase
183	76	3	109	15	zinc finger protein
265	52	1	2437	52	UDP-glucuronosyltransferase
297	37	1	1093	36	antichymotrypsin
702	17	1	855	17	spermine oxidase
2855	15	1	536	15	uncharacterized protein
5863	49	1	74	7	zinc finger protein
Total	289	9	6452	185	
